# Apatinib in patients with recurrent or metastatic thymic epithelial tumor: a single-arm, multicenter, open-label, phase II trial

**DOI:** 10.1186/s12916-022-02361-w

**Published:** 2022-05-10

**Authors:** Zhengbo Song, Guangyuan Lou, Yina Wang, Zhiping Yang, Wenxian Wang, Yongling Ji, Shiqing Chen, Chunwei Xu, Xiao Hu, Yiping Zhang

**Affiliations:** 1 Department of Clinical Trial, The Cancer Hospital of the University of Chinese Academy of Sciences (Zhejiang Cancer Hospital), Hangzhou, 310022 Zhejiang China; 2 Department of Medical Oncology, The Cancer Hospital of the University of Chinese Academy of Sciences (Zhejiang Cancer Hospital), Hangzhou, 310022 Zhejiang China; 3 Department of Medical Oncology, The First Affiliated Hospital, College of Medicine, Zhejiang University, Hangzhou, 310000 Zhejiang China; 4 Department of Medical Oncology, The First Hospital of Jiaxing, Jiaxing, 314000 Zhejiang China; 5 Department of Radiotherapy Oncology, The Cancer Hospital of the University of Chinese Academy of Sciences (Zhejiang Cancer Hospital), Hangzhou, 310022 Zhejiang China; 6 The Medical Department, 3D Medicines Inc., Shanghai, 201114 China; 7 Department of Respiratory Medicine, Jinling Hospital, Nanjing University School of Medicine,Nanjing, 210002,, Jiangsu, China

**Keywords:** Thymic epithelial tumors, Apatinib, Efficacy, Toxicities

## Abstract

**Background:**

Thymic epithelial tumors (TETs) are rare malignancies and the treatment options are limited. We aimed to evaluate the efficacy and safety of apatinib, an angiogenesis inhibitor, in advanced TETs.

**Methods:**

This was an open-label, single-arm, phase II trial at three centers in China. Patients with TET who had progressed after failure of at least one line of platinum-based chemotherapy were enrolled. Patients received apatinib 500 mg orally per day. The primary endpoint was objective response rate (ORR). Secondary endpoints were progression-free survival (PFS), overall survival (OS), disease control rate (DCR), and safety.

**Results:**

From June 29, 2017, to April 18, 2019, 25 patients were enrolled. At data cut off (September 30, 2021), one patient achieved complete response, nine achieved partial response, and 11 achieved stable disease, with an ORR of 40% (95% CI 21–61%) and DCR of 84% (95% CI 64–95%). The median PFS was 9.0 (95% CI 5.4–12.6) months. The median OS was 24.0 (95% CI 8.2–39.8) months. All patients reported treatment-related adverse events (TRAEs). Grade 3 TRAEs occurred 26 times in 15 patients. No grade 4 or 5 toxicities occurred.

**Conclusions:**

This is the first trial of apatinib for the treatment of TETs. Apatinib showed promising antitumor activity and the toxicities were tolerable and manageable.

**Supplementary Information:**

The online version contains supplementary material available at 10.1186/s12916-022-02361-w.

## Background

Thymic epithelial tumors (TETs), consisting of thymoma (T) and thymic carcinoma (TC), are rare malignancies in adults. T typically induces a multitude of autoimmune diseases, whereas TC is usually more aggressive and associated with distant metastases, resulting in a dismal prognosis (5-year survival rate of 30–50% for stage IV patients) [1]. A report from the European Society of Thoracic Surgeons (ESTS) prospective thymic database showed that compared to ESTS retrospective database, the prevalence of TCs has increased from 9 to 28% [2]. The prognosis was good for patients who were eligible for surgical resection. Our previous retrospective study showed that the 5-year disease-free survival rate and overall survival (OS) rate for TCs after resection were 59.7% and 66.2%, respectively [3]. However, about 10–15% of patients eventually developed tumor recurrence, and even the World Health Organization (WHO) type A T was reported to experience metastasis or recurrence [4, 5]. For unresectable or metastatic disease, the systemic therapy is an important issue. Platinum-based combination chemotherapy exhibiting good response and survival benefit is the standard regimen as first-line therapy, and studies showed that there was no significant difference in efficacy between first-line chemotherapy regimens [6].^.^ For patients who failed first-line chemotherapy, pemetrexed-based regimen, paclitaxel plus carboplatin, and docetaxel-based chemotherapy could only achieve a median progression-free survival (PFS) of 3–4 months [6–8]. Until now, there are few available treatment options after failure of platinum-based chemotherapy. The analysis of genetic variability for TETs showed no specific hotspot gene mutation, which might explain the unideal results of targeted therapies for TETs in a previous report [9]. Immunotherapy for TETs has also showed moderate anti-tumor activity [10, 11], but patients may have a very long duration of response if they are sensitive to immunotherapy [12]. Nevertheless, careful selection of patients and monitoring of immune-related adverse events (AEs) with higher than expected incidence are warranted.

Molecular aberrations in TETs and target treatment approaches are always investigated. Studies have showed that vascular endothelial growth factor (VEGF) and its receptors (VEGFR-1, VEGFR-2, and VEGFR-3) are overexpressed in high-risk TETs [13, 14], and there appears a distinct association between VEGF and invasiveness [15]. Serum VEGF and basic fibroblast growth factor levels are significantly high in patients with TETs [16]. A few phase II trials have demonstrated that antiangiogenic treatments (such as bevacizumab, sunitinib, regorafenib, and lenvatinib) may have clinical benefits in TETs [17–20]. Several case reports suggested the activity of sorafenib in TETs [21–24].

Apatinib, an oral angiogenesis inhibitor targeting VEGFR-2, has been approved for advanced hepatocellular carcinoma and adenocarcinoma of the stomach or gastroesophageal junction in China [25–27]. Two case reports reported the anti-tumor activity of apatinib in patients with advanced TC. Both patients presented partial response (PR) [28, 29], suggesting that apatinib may be an alternative treatment option for TETs after chemotherapy failure.

Based on these considerations, we conducted this study to investigate the efficacy and safety of apatinib in patients with TETs after failure of platinum-based chemotherapy.

## Methods

### Study design and patients

This study was an open-label, single-arm, phase II trial conducted at three centers (Zhejiang Cancer Hospital, The First Affiliated Hospital, College of Medicine, Zhejiang University and The First Hospital of Jiaxing) in China. Patients were included if they were aged ≥18 years, had pathologically confirmed T or TC, had progressed after at least one line of systemic therapy with platinum-based chemotherapy, had an Eastern Cooperative Oncology Group (ECOG) performance status of 0–2, had a life expectancy of at least 3 months, and had an adequate hepatic and renal function. The key exclusion criteria were previous exposure to targeted therapy (including recombinant human endostatin, bevacizumab, and single- or multi-target tyrosine kinase inhibitors), active brain metastases, or other malignancies.

The study protocol (No. IRB-2017-13) was approved by the institutional ethics committee of Zhejiang Cancer Hospital, the institutional ethics committee of The First Affiliated Hospital, College of Medicine, Zhejiang University, and the institutional ethics committee of The First Hospital of Jiaxing, respectively. Written informed consent was obtained from all patients before enrollment. This trial was registered with Chictr.org.cn, number ChiCTR-ONC-17013108.

#### Treatment procedures

Patients received oral apatinib 500 mg once daily until disease progression, unacceptable toxicities, or withdrawal of consent. Each 28 days were deemed as a treatment cycle. When grade ≥3 AEs occurred, apatinib treatment would be interrupted. When grade ≥3 AEs recovered to grade ≤1, apatinib treatment could be resumed with a dose reduction. In the whole treatment duration, the dose reduction was allowed no more than twice, reaching a dose level of 250 mg per day, and further dose reduction was not permitted. Treatment interruption caused by toxicities could not exceed 14 days (either continuously or cumulatively) and was allowed no more than twice in a treatment cycle. If the treatment interruption exceeded 14 days, apatinib would be discontinued.

At the end of every two cycles, a computed tomography or magnetic resonance imaging evaluation according to the Response Evaluation Criteria In Solid Tumors (RECIST) version 1.1 was performed to evaluate the response of tumors. Complete response (CR) and PR would be confirmed four weeks later. AEs were recorded from baseline until at least 28 days after the last dose of the study drug was administered, and graded according to the National Cancer Institute Common Terminology Criteria for Adverse Events version 5.0.

#### Endpoints

The primary endpoint was confirmed objective response rate (ORR), defined as the proportion of patients with the best response of CR or PR, as assessed by the investigator. Secondary endpoints were PFS, OS, disease control rate (DCR), and safety. PFS was defined as the time from the initiation of apatinib treatment to disease progression or death from any cause, whichever occurred first. OS was defined as the time from the initiation of apatinib treatment to death from any cause. DCR was defined as the proportion of patients with the best response of CR, PR, or stable disease (SD).

#### Statistical analysis

We conducted a single-arm, phase II trial with the confirmed ORR as the primary endpoint. Twenty-five patients would provide 80% power to detect an ORR rate of 25% at a 0.5% alpha level under the null hypothesis of ORR = 10%.

Patients who received at least one cycle of apatinib treatment were included for efficacy and safety analyses. Efficacy was analyzed in the overall population and subgroup according to the histology (T and TC). PFS and OS were estimated using the Kaplan-Meier method and compared using the log-rank test. The 95% confidence intervals (CIs) of survival were calculated using the Brookmeyer-Crowley method. DCR and ORR were compared using Fisher’s exact test. The 95% CIs of DCR and ORR were calculated using the Clopper-Pearson method. Statistical analyses were performed using SAS version 9.2. *P* < 0.05 was considered statistically significant.

## Results

### Patient and disease characteristics

**Fig. 1:**
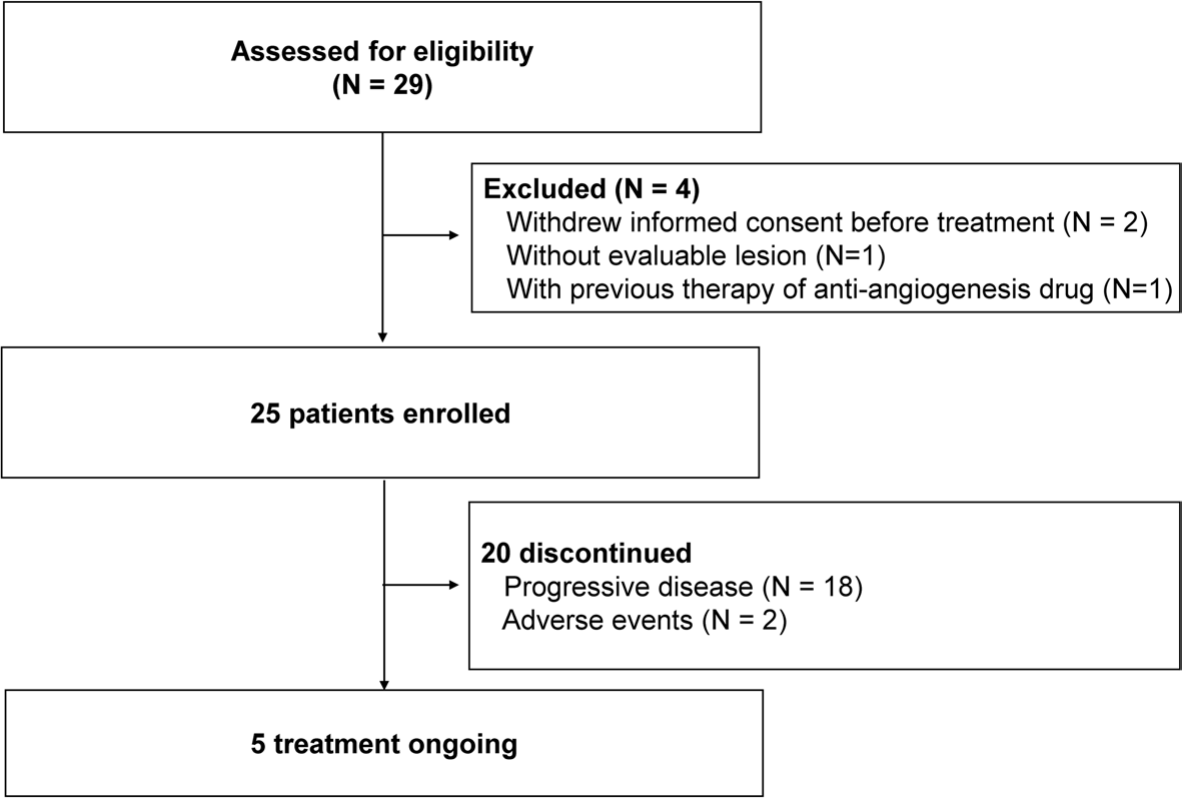
Study flowchart

From June 29, 2017, to April 18, 2019, a total of 29 patients were assessed for eligibility (Fig. 1). Four patients were excluded from the study, including two patients who withdrew the informed consent before treatment, one without evaluable lesion, and one with previous therapy of anti-angiogenesis drug. Twenty-five patients received at least one cycle of apatinib treatment and were evaluable for efficacy and safety. Of 25 patients, the median age was 53 (range, 26–70) years, and 17 (68%) were males. There were four (16%) patients in Masaoka’s stage IVa and 21 (84%) in stage IVb. Of ten patients with T, one had AB, two had B2, two had B3, and five had mixed histological features. Of 15 patients with TC, seven were squamous cell carcinoma, two were adenocarcinoma, and six were poorly differentiated with other types of TC. The ECOG performance status was 0 (4 [16%]) or 1 (21 [75%]) for all 25 patients. The median number of previous therapy line was 2 (range, 1–5). Most (20 [80%]) patients had ≥2 metastatic lesions. Seven (28%) patients had liver metastases. The details of patient characteristics are shown in Table 1.

**Table 1:** Baseline characteristics of patients with thymic epithelial tumor

Characteristic	Apatinib (*n* = 25)
Sex, *n* (%)	
Male	17 (68)
Female	8 (32)
Age, years	
Median (range)	53 (26–70)
≥60, *n* (%)	7 (28)
<60, *n* (%)	18 (72)
Smoking history, *n* (%)	
Yes	10 (40)
No	15 (60)
Histology, *n* (%)	
Thymic carcinoma	15 (60)
Thymoma	10 (40)
Masaoka’s stage, *n* (%)	
IVa	4 (16)
IVb	21 (84)
Number of prior therapy lines	
1, *n* (%)	12 (48)
≥2, *n* (%)	13 (52)
ECOG performance status, *n* (%)	
0	4 (16)
1	21 (84)
Number of metastatic lesions, *n* (%)	
1	5 (20)
≥2	20 (80)

*ECOG* Eastern Cooperative Oncology Group

### Efficacy

As of September 30, 2021, five of 25 patients were still receiving apatinib treatment without tumor progression. Twenty patients discontinued treatment due to tumor progression (n = 18) and AEs (*n* = 2). Common tumor progression sites included lung (*n* = 7), mediastinum (*n* = 5), liver (*n* = 3), pleura (*n* = 3), bone (*n* = 2), and others (*n* = 5).

**Fig. 2:**
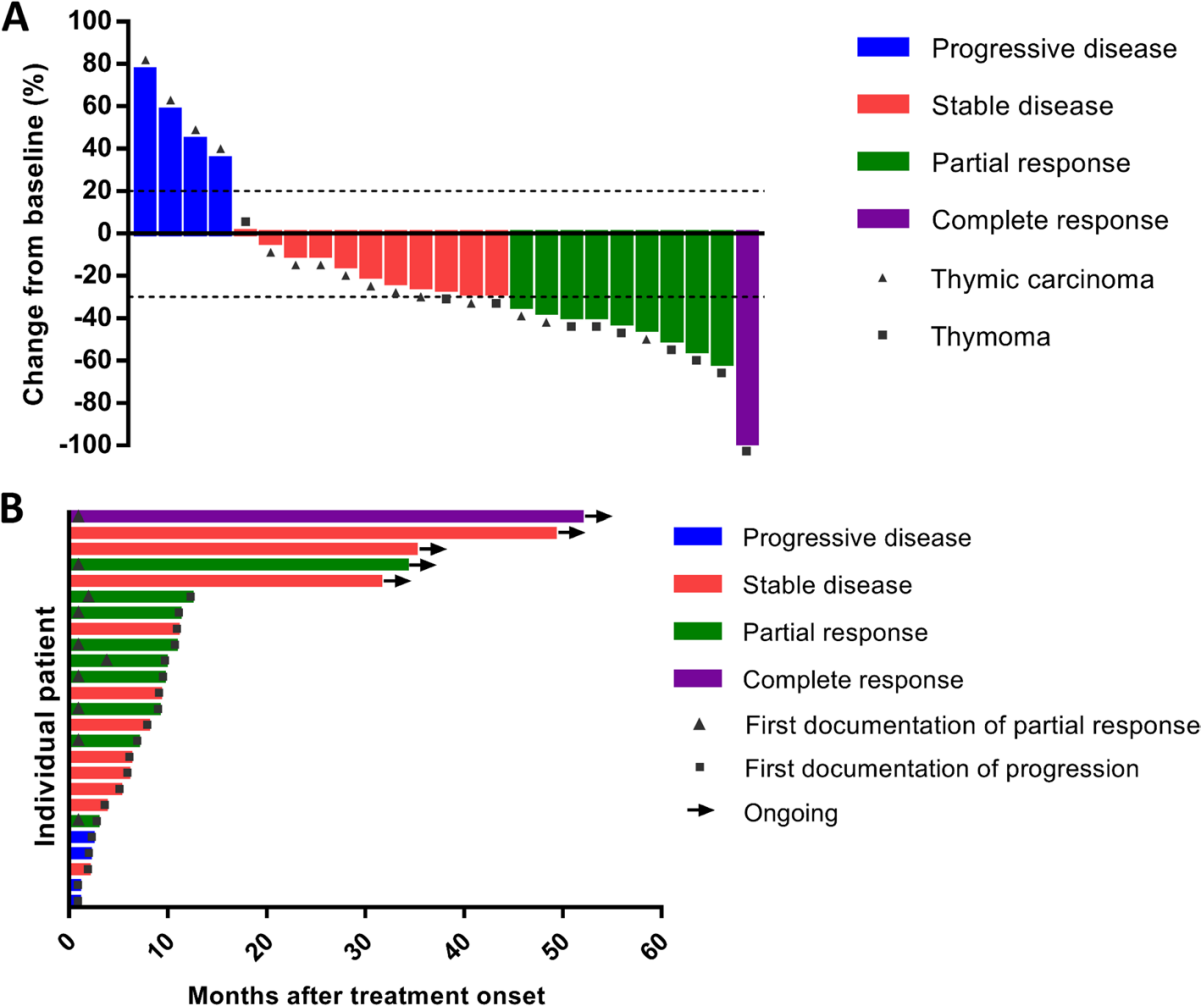
**A** Waterfall plot for best volumetric response to apatinib. **B** Swimming plot for progression-free survival

**Fig. 3:**
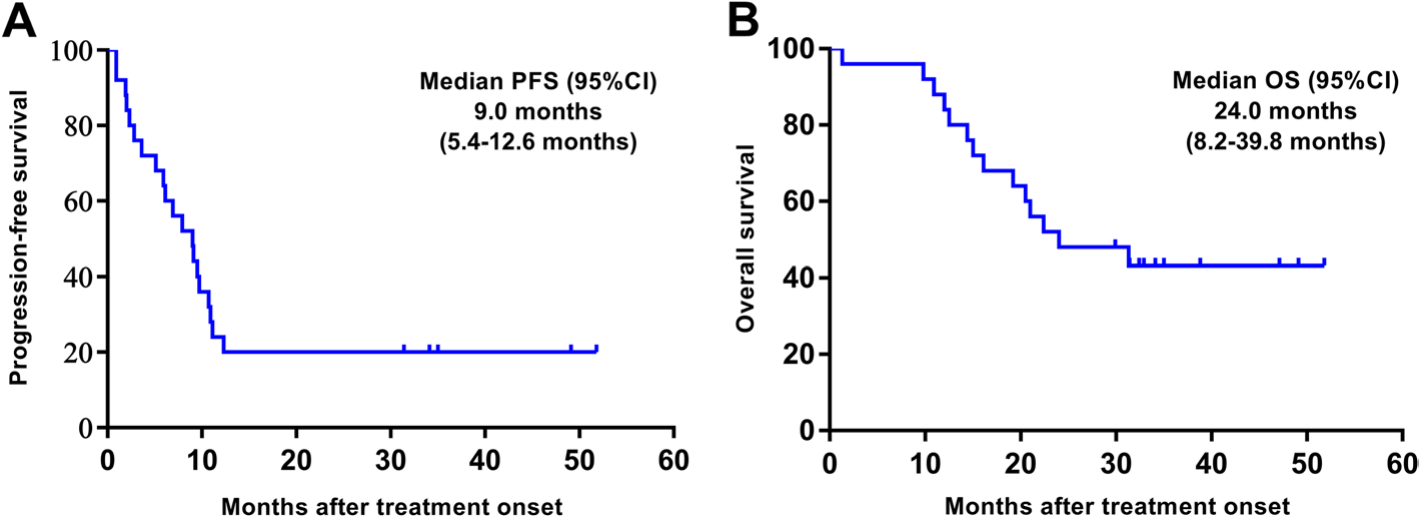
The Kaplan–Meier estimates of **A** progression-free survival and **B** overall survival

One patient achieved CR and nine patients achieved PR, with an ORR of 40% (95% CI 21–61%). Eleven patients achieved SD, with a DCR of 84% (95% CI 64–95%) (Fig. 2A). The ORR and DCR in ten patients with T were 70% (95% CI 35–93%) and 100% (95% CI 69–100%), respectively. The ORR and DCR in 15 patients with TC were 20% (95% CI 4–48%) and 73% (95% CI 45–92%), respectively. Details of individual patient characteristics and best tumor response were summarized in Additional file 1: Table S1. The swimming plot for PFS is shown in Fig. 2B. The median PFS was 9.0 (95% CI 5.4–12.6) months in all 25 patients (Fig. 3A), 9.5 (95% CI 8.6–10.4) months in ten patients with T, and 6.1 (95% CI 2.6–9.6) months in 15 patients with TC. The median OS was 24.0 (95% CI 8.2–39.8) months in all 25 patients (Fig. 3B), 22.4 (95% CI 6.4–38.4) months in ten patients with T, and 24.0 (95% CI 16.1–∞) months in 15 patients with TC.

**Fig. 4:**
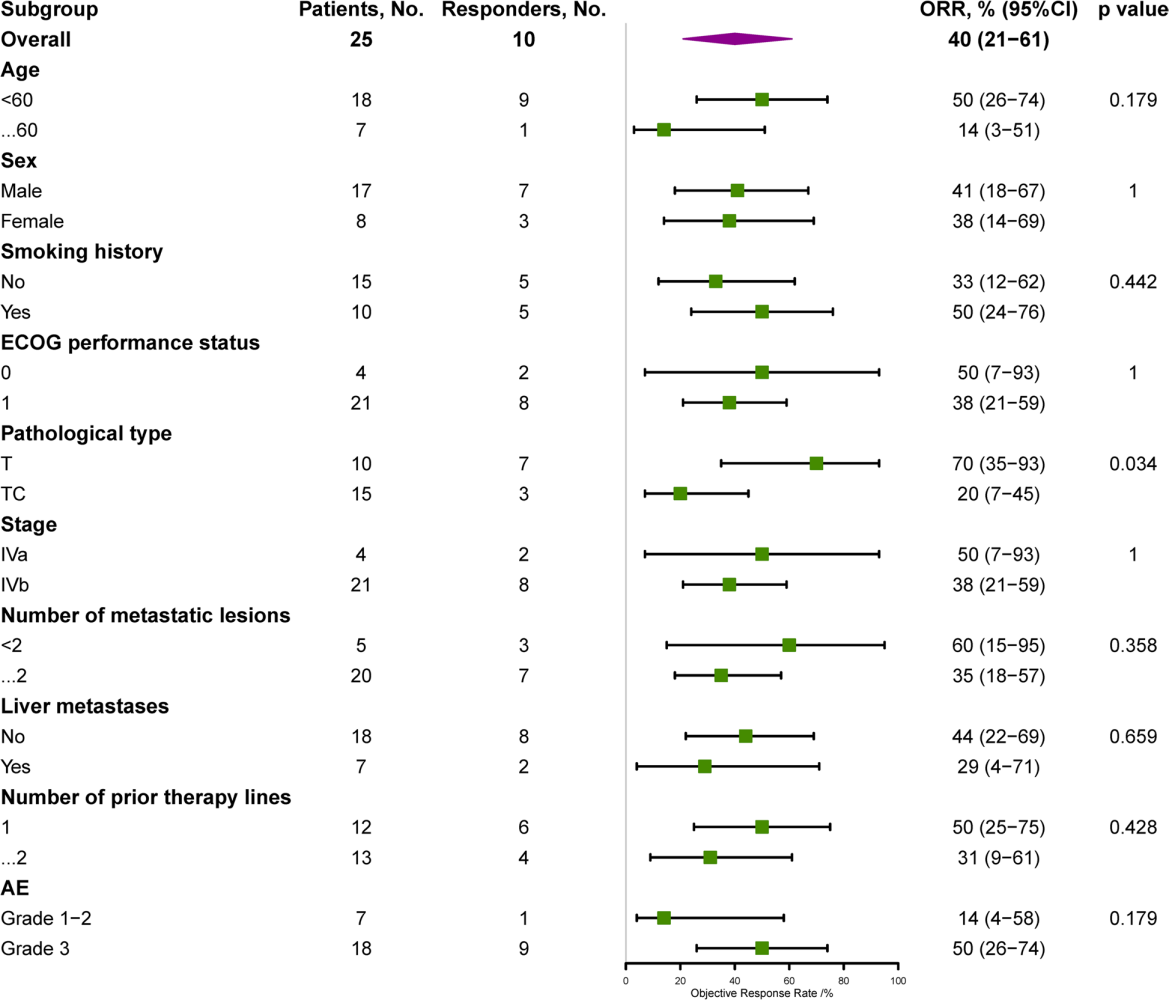
Forest plots of hazard ratios in key subgroups for an objective response rate

In addition, we explore the association between baseline characteristics and ORR (Fig. 4), PFS (Additional file 1: Fig. S1), and OS (Additional file 1: Fig. S2). The baseline characteristics included sex, age, smoking history, stage, pathological type, ECOG performance status, number of metastatic lesions, the absence or presence of liver metastasis, number of previous treatment lines, and AEs. Except for pathological type, no baseline characteristic was associated with ORR (Fig. 4). The median PFS of patients with grade ≥3 AEs was significantly longer than that of patients without grade ≥3 AEs (9.7 vs. 3.6 months, hazard ratio, 0.29; 95% CI, 0.11−0.77; *p* = 0.008) (Additional file 1: Fig. S1).

### Safety

All of the 25 patients (100%) reported treatment-related AEs (TRAEs) and most were with grade 1–2. Grade 3 TRAEs occurred 26 times in 15 patients. The most common grade 3 TRAEs included hypertension (8 [32%]), hand-foot syndrome (5 [20%]), proteinuria (3 [12%]), lymphocytopenia (3 [12%]), fatigue (2 [8%]), nausea (1 [4%]), vomiting (1 [4%]), oral mucositis (1 [4%])), gamma-glutamyltransferase increased (1 [4%]), and neutrophilic granuloaytopenia (1 [4%]) (Table 2). No grade 4 or 5 AEs were recorded. A total of 18 patients underwent dose reduction to 250 mg/day due to AEs. Two patients discontinued apatinib treatment owing to AEs.

**Table 2:** Treatment-related adverse events occurring in ≥10% of patients

Adverse event	All grades ^a^	Apatinib (*n* = 25), *n* (%)		
		Grade 1	Grade 2	Grade 3
Fatigue	22 (88)	13 (52)	7 (28)	2 (8)
Hand-foot syndrome	20 (80)	5 (20)	10 (40)	5 (20)
AST increased	17 (68)	16 (64)	1 (4)	
Proteinuria	16 (64)	7 (28)	6 (24)	3 (12)
Thrombocytopenia	16 (64)	13 (52)	3 (12)	
Diarrhea	16 (64)	9 (36)	7 (28)	
Headache	15 (60)	13 (52)	2 (8)	
Nausea	14 (56)	10 (40)	3 (12)	1 (4)
Decreased appetite	14 (56)	11 (44)	3 (12)	
Dizziness	14 (56)	13 (52)	1 (4)	
Hypertension	13 (52)		5 (20)	8 (32)
Urine occult blood	12 (48)	11 (44)	1 (4)	
Anemia	12 (48)	7 (28)	5 (20)	
ALT increased	11 (44)	9 (36)	2 (8)	
Mucositis oral	10 (40)	5 (20)	4 (16)	1 (4)
WBC decreased	10 (40)	6 (24)	4 (16)	
Blood bilirubin increased	10 (40)	7 (28)	3 (12)	
Hyponatremia	10 (40)	10 (40)		
Vomiting	10 (40)	7 (28)	2 (8)	1 (4)
Neutrophilic granuloaytopenia	9 (36)	5 (20)	3 (12)	1 (4)
Cough	8 (32)	8 (32)		
Creatinine increased	7 (28)	5 (20)	2 (8)	
Hypochloremia	5 (20)	5 (20)		
Lymphocytopenia	5 (20)	1 (4)	1 (4)	3 (12)
Weight loss	5 (20)	5 (20)		
Chest distress	5 (20)	4 (16)	1 (4)	
Hoarseness	4 (16)	4 (16)		
Hypertriglyceridemia	4 (16)	4 (16)		
GGT increased	3 (12)		2 (8)	1 (4)
Constipation	3 (12)	3 (12)		

^a^No grade 4 or 5 adverse events occurred

*ALT* alanine transaminase, *AST* aspartate transferase, *GGT* gamma-glutamyltransferase, *WBC* white blood cell count

## Discussion

To our knowledge, this is the first study to prospectively demonstrate the robust and durable clinical benefits of apatinib in heavily pretreated patients with thymic malignancies. We observed that apatinib achieved an ORR of 40% (T: 70%; TC: 20%) and a DCR of 84% (T: 100%; TC: 73%) in patients with TETs, including 52% heavily treated patients who had received two or more lines of prior systemic therapy. The median PFS and OS were 9.0 (T: 9.5; TC: 6.1) months and 24.0 (T: 22.4; TC: 24.0) months, respectively. Moreover, apatinib showed a favorable tolerability and safety for patients with T and TC.

We reviewed the studies published in recent years which investigated targeted therapy in advanced TETs, summarized in Additional file 1: Table S2. In a phase II trial of cixutumumab, the results showed an ORR of 0%, DCR of 42%, and median PFS of 1.7 months in 12 patients with TC, whereas 37 patients with T showed an ORR of 14%, DCR of 89%, and median PFS of 9.9 months [30]. In another phase II trial of 50 patients (32 T and 18 TC) treated with everolimus, the ORR was 17% in TC and 9% in T, respectively [31]. Sunitinib exhibited a good therapeutic effect and has been recommended by National Comprehensive Cancer Network guidelines as the second-line standard treatment for advanced TC. Previous phase II data of sunitinib showed that in 23 assessable patients with TC, the ORR was 26% and the median PFS was 7.2 months [18]. Of 16 patients with T, the ORR with sunitinib was 6% and the median PFS was 8.5 months [18]. Recently, the immune checkpoint inhibitors (pembrolizumab and nivolumab) have also been investigated in previously treated, advanced T and TC. A phase II trial of pembrolizumab showed an ORR of 19% and 29% in TC and T, respectively [11]. Nivolumab was also examined in a cohort of previously treated TC but the results showed no patients with objective response, with a DCR of 79% and median PFS of 3.8 months [32]. In our study, apatinib showed comparable efficacy with sunitinib, with a high tumor response rate and long survival benefit in T. Despite the different clinical settings among studies, the indirect comparisons suggested that apatinib was worthy of further investigation in advanced TETs.

In terms of safety, major AEs in this study were hypertension, hand-foot syndrome, and proteinuria, which were consistent with the toxicity profile of apatinib reported in large-scale clinical trials [26, 27]. Grade 3 TRAEs were observed in 15 patients and no grade 4 or 5 AEs occurred. In the study of sunitinib, the most common AEs were grade 3 or higher lymphocytopenia, fatigue, and oral mucositis, and the cardiotoxicity of sunitinib led to a decrease in left ventricular ejection fraction and death [18]. Everolimus showed the potential high risk of fatal pneumonitis [34]. Compared with those targeted drugs, apatinib showed less toxicities. In addition, the immune checkpoint inhibitor pembrolizumab showed a high incidence of immune-related AEs [10, 11]. Overall, the toxicities of apatinib in our study were tolerable and manageable.

The major limitations of our study are the single-arm design and the relatively small sample size due to the rarity of patients with TETs. However, this prospective trial showed the potential antitumor activity of apatinib in patients with refractory TETs.

## Conclusions

This study was the first prospective trial of apatinib in patients with advanced TETs. Apatinib showed encouraging antitumor activity and a well-tolerated safety profile in refractory or relapsed TETs, making it an alternative treatment option for patients with advanced TETs.

## Supplementary Information

**Additional file 1: Table S1, Figure S1-S2. Table S1.** Efficacy of Different Targeted Therapy in Thymic Epithelial Tumors. **Figure S1.** The association between baseline characteristics and progression free survival. **Figure S2.** The association between baseline characteristics and overall survival.

## Data Availability

The datasets used and/or analyzed during the current study are available from the corresponding author upon reasonable request.

## Abbreviations

AE: Adverse event
CI: Confidence interval
CR: Complete response
DCR: Disease control rate
ECOG: Eastern Cooperative Oncology Group
ESTS: European Society of Thoracic Surgeons
ORR: Objective response rate
OS: Overall survival
PFS: Progression-free survival
PR: Partial response
RECIST: Response Evaluation Criteria In Solid Tumors
SD: Stable disease
T: Thymoma
TC: Thymic carcinoma
TET: Thymic epithelial tumor
TRAE: Treatment-related adverse event
VEGF: Vascular endothelial growth factor
VEGFR: Vascular endothelial growth factor receptor
WHO: World Health Organization
