# Supplementary material for: Apatinib in patients with recurrent or metastatic thymic epithelial tumor: a single-arm, multicenter, open-label, phase II trial
Source: BMC Med. 2022 May 10;20:154. doi: 10.1186/s12916-022-02361-w (PMC9088066; doi:10.1186/s12916-022-02361-w)
Supplement: Supplementary file 1 — Additional file 1: Table S1, Figure S1-S2. Table S1. Efficacy of Different Targeted Therapy in Thymic Epithelial Tumors. Figure S1. The association between baseline characteristics and progression free survival. Figure S2. The association between baseline characteristics and overall survival. [file 12916_2022_2361_MOESM1_ESM.docx]

**Supplementary tables and figures**

**Table S1.** Individual patient characteristics and best tumor response

| Patient number | Age (years) | Sex | Histology | Stage | Metastatic site | Number of prior therapy lines | Apatinib treatment duration (months) | Maximum tumor size change (%) | Best tumor response |
| --- | --- | --- | --- | --- | --- | --- | --- | --- | --- |
| 1 | 38 | male | T | IVa | pleura | 2 | 51.8 | -100 | CR |
| 2 | 56 | female | T | IVb | pleura, lung | 2 | 6.9 | -42 | PR |
| 3 | 56 | male | T | IVb | pleura, mediastinum | 2 | 2.8 | -55 | PR |
| 4 | 41 | male | T | IVb | pleura, mediastinum, liver | 2 | 9.1 | -26 | SD |
| 5 | 70 | female | T | IVb | lung, liver | 2 | 10.7 | -61 | PR |
| 6 | 63 | male | T | IVb | pleura, lung | 2 | 35 | -28 | SD |
| 7 | 50 | male | T | IVb | lung, bone | 1 | 9.7 | -39 | PR |
| 8 | 49 | male | T | IVa | pleura | 1 | 9.5 | -50 | PR |
| 9 | 50 | female | T | IVb | pleura, liver, brain | 2 | 3.6 | 0.5 | SD |
| 10 | 44 | male | T | IVb | pleura, lung | 1 | 34.1 | -39 | PR |
| 11 | 60 | male | TC | IVa | pleura | 1 | 0.9 | 58 | PD |
| 12 | 50 | male | TC | IVb | pleura, mediastinum, lung | 2 | 49.1 | -28 | SD |
| 13 | 53 | male | TC | IVb | pleura, mediastinum, bone | 1 | 11.1 | -45 | PR |
| 14 | 56 | male | TC | IVb | mediastinum, lung | 2 | 1.9 | -4 | SD |
| 15 | 53 | female | TC | IVb | pleura, mediastinum, lung | 2 | 5.1 | -10 | SD |
| 16 | 49 | male | TC | IVb | lung, bone | 1 | 9 | -37 | PR |
| 17 | 63 | male | TC | IVb | pleura, mediastinum, lung | 1 | 6.1 | -25 | SD |
| 18 | 60 | female | TC | IVb | mediastinum, lung | 1 | 5.9 | -15 | SD |
| 19 | 63 | male | TC | IVb | liver | 1 | 31.4 | -23 | SD |
| 20 | 26 | female | TC | IVb | liver | 1 | 12.3 | -34 | PR |
| 21 | 55 | female | TC | IVb | lung, liver | 1 | 10.9 | -10 | SD |
| 22 | 55 | male | TC | IVb | pleura, bone | 2 | 7.9 | -20 | SD |
| 23 | 49 | male | TC | IVb | pleura, mediastinum | 1 | 2 | 44 | PD |
| 24 | 62 | male | TC | IVb | lung, liver | 2 | 0.9 | 77 | PD |
| 25 | 40 | female | TC | IVa | pleura | 2 | 2.3 | 35 | PD |

CR: complete response; PD: progressive disease; PR: partial response; SD: stable disease; T: thymoma; TC: thymic carcinoma.

**Table S2.** Efficacy of Different Targeted Therapy in Thymic Epithelial Tumors.

| Article | Therapy | Therapy line | Number of patients | ORR | DCR | mPFS (months) | mOS (months) |
| --- | --- | --- | --- | --- | --- | --- | --- |
| Rajan 2014^32^  All (T/TC)  T  TC | Cixutumumab | ≥2 | 49  37  12 | 10%  14%  0% | 78%  89%  42% | 8.2  9.9  1.7 | 16.2  27.5  8.4 |
| Gubens 2015^37^  All (T/TC)  T  TC | Saracatinib | ≥2 | 21  12  9 | 0%  0%  0% | 43%  67%  11% | 2.5  5.3  0.9 | 23.1  37.5  6.7 |
| Thomas 2015^17^  All (T/TC)  T  TC | Sunitinib | ≥2 | NA  16  23 | NA  6%  26% | NA  81%  91% | NA  8.5  6.7 | NA  NR  16.3 |
| Remon 2016^38^  All (T/TC)  T  TC | Sunitinib | ≥4 | 28  8  20 | 21%  25%  20% | 61%  75%  55% | 3.7  5.4  3.3 | 15.4  NR  12.3 |
| Zucali 2017^33^  All (T/TC)  T  TC | Everolimus | ≥2 | 50  32  18 | 12%  9%  17% | 88%  94%  78% | 10.1  16.6  5.6 | 25.7  NR  14.7 |
| Matteo 2018^18^  All (T/TC)  T  TC | Regorafenib | ≥2 | 19  7  12 | 5%  14%  0% | 79%  NR  NR | 8.9  NA  NA | NR  NR  NR |
| Giaccone 2018^11^  All (T/TC)  T  TC | Pembrolizumab | ≥2 | 40  NA  40 | 23%  NA  23% | 75%  NA  75% | 4.2  NA  4.2 | 24.9  NA  24.9 |
| Cho 2018^10^  All (T/TC)  T  TC | Pembrolizumab | ≥2 | 33  7  26 | 21%  29%  19% | 79%  100%  73% | 6.1  6.1  6.1 | NR  NR  14.5 |
| KatsuyaYuki 2019^34^  All (T/TC)  T  TC | Nivolumab | ≥2 | 15  NA  15 | 0%  NA  0% | 79%  NA  79% | 3.8  NA  3.8 | NR  NA  NR |

DCR, disease control rate; T, thymoma; TC, thymic carcinoma; mOS, median overall survival; mPFS, median progression-free survival; NA, not available; NR, not reached; ORR, objective response rate.

**Figure S1.** The association between baseline characteristics and progression free survival.


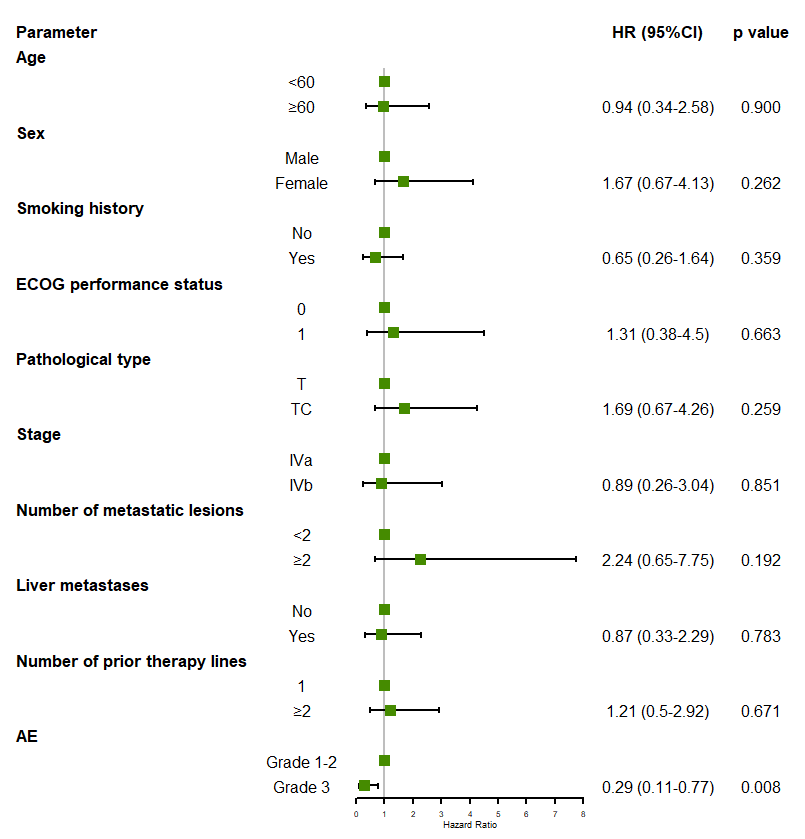


**Figure S2.** The association between baseline characteristics and overall survival.

**
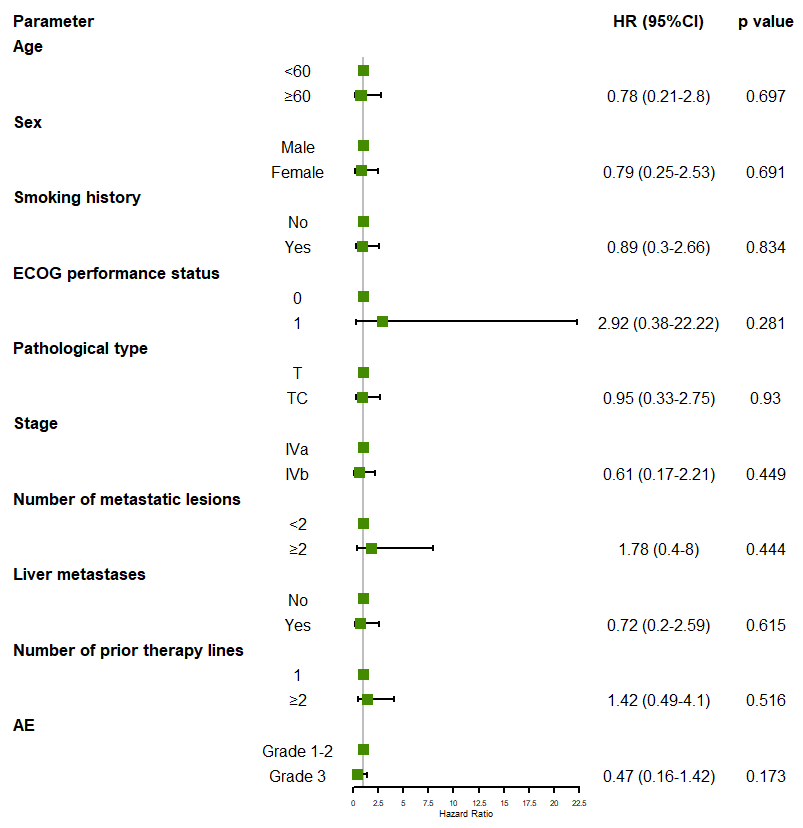
**
